# Core–shell structured gold nanoparticles as carrier for ^166^Dy/^166^Ho in vivo generator

**DOI:** 10.1186/s41181-022-00170-3

**Published:** 2022-07-19

**Authors:** Runze Wang, Bernard Ponsard, Hubert Wolterbeek, Antonia Denkova

**Affiliations:** 1 Applied Radiation and Isotopes, Department of Radiation Science and Technology, Faculty of Applied Sciences, Delft University of Technology, Mekelweg 15, 2629 JB Delft, The Netherlands; 2 Belgian Nuclear Research Centre, SCK CEN, Boeretang 200, 2400 Mol, Belgium

**Keywords:** Radionuclide therapy, Dysprosium-166, Holmium-166, In vivo generator, Internal conversion, Gold nanoparticle

## Abstract

**Background:**

Radionuclide therapy (RNT) has become a very important treatment modality for cancer nowadays. Comparing with other cancer treatment options, sufficient efficacy could be achieved in RNT with lower toxicity. β^−^ emitters are frequently used in RNT due to the long tissue penetration depth of the β^−^ particles. The dysprosium-166/holmium-166 (^166^Dy/^166^Ho) in vivo generator shows great potential for treating large malignancies due to the long half-life time of the mother nuclide ^166^Dy and the emission of high energy β^−^ from the daughter nuclide ^166^Ho. However, the internal conversion occurring after β^−^ decay from ^166^Dy to ^166^Ho could cause the release of about 72% of ^166^Ho when ^166^Dy is bound to conventional chelators. The aim of this study is to develop a nanoparticle based carrier for ^166^Dy/^166^Ho in vivo generator such that the loss of the daughter nuclide ^166^Ho induced by internal conversion is prevented. To achieve this goal, we radiolabelled platinum-gold bimetallic nanoparticles (PtAuNPs) and core–shell structured gold nanoparticles (AuNPs) with ^166^Dy and studied the retention of both ^166^Dy and ^166^Ho under various conditions.

**Results:**

The ^166^Dy was co-reduced with gold and platinum precursor to form the ^166^DyAu@AuNPs and ^166^DyPtAuNPs. The ^166^Dy radiolabelling efficiency was determined to be 60% and 70% for the two types of nanoparticles respectively. The retention of ^166^Dy and ^166^Ho were tested in MiliQ water or 2.5 mM DTPA for a period of 72 h. In both cases, more than 90% of both ^166^Dy and ^166^Ho was retained. The results show that the incorporation of ^166^Dy in AuNPs can prevent the escape of ^166^Ho released due to internal conversion.

**Conclusion:**

We developed a chelator-free radiolabelling method for ^166^Dy with good radiolabelling efficiency and very high stability and retention of the daughter nuclide ^166^Ho. The results from this study indicate that to avoid the loss of the daughter radionuclides by internal conversion, carriers composed of electron-rich materials should be used.

**Supplementary Information:**

The online version contains supplementary material available at 10.1186/s41181-022-00170-3.

## Introduction

Cancer is one of the leading causes of death in the world (Sung et al. 2021; Bray et al. 2018). Nowadays, surgery and external beam radiation therapy (EBRT) are still the most common treatment modalities for localized tumors. In the case of metastases, systemic treatments such as radionuclide therapy (RNT) are preferred. RNT has been proved to be able to significantly prolong the life expectancy of terminal patients without affecting quality of life (Pool et al. 2010; Humm et al. 2015). In RNT, the therapeutic radionuclides are usually linked to chelators conjugated to tumor targeting vectors such as peptides, nucleotides and antibodies. Once distributed to the tumor site, the ionizing radiation emitted by the radionuclides can damage the DNA of the cancer cells and lead to apoptosis (Gudkov et al. 2016; Dash et al. 2013; Sgouros et al. 2020; Tafreshi et al. 2019; Kostelnik and Orvig 2019).

Over the past decades, many radiopharmaceuticals have been developed and some of them have been already applied in the clinic (Suman et al. 2021). Radionuclides that emit β^−^ particles are more commonly applied in the clinic but the interest in α emitters is also growing (Nelson et al. 2021; Tafreshi et al. 2019). Since β^−^ particles have relatively long tissue penetration depth, they are suitable for treating larger metastases (Pouget et al. 2011; Marcu et al., 2018). Moreover, additional benefits can be achieved with β^−^ emitters by the so called “cross-fire” effect, i.e. due to the long range of β^−^ particles, it is not essential to target every single tumor cell to efficiently irradiate the whole tumor (Pouget et al. 2011).

**Fig. 1:**
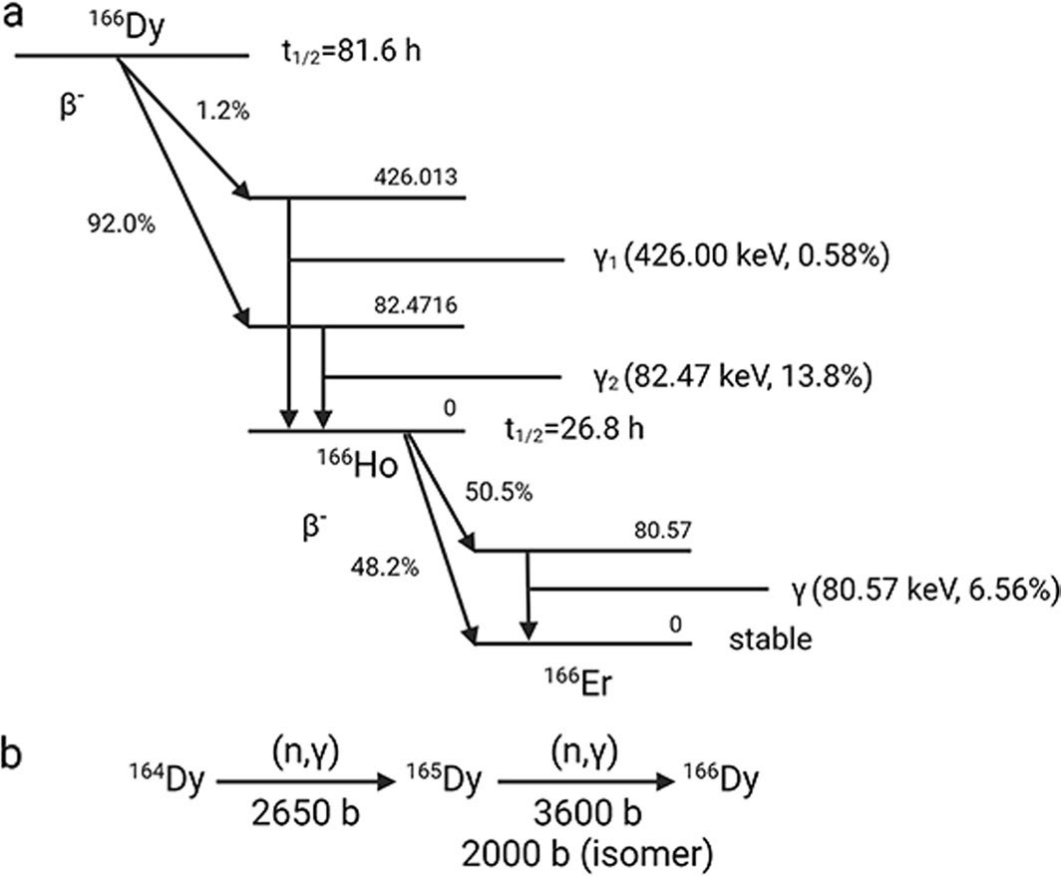
**a** Decay scheme of ^166^Dy and ^166^Ho including the major transitions. **b** The double-neutron capture nuclear reaction of ^164^Dy to produce ^166^Dy and the corresponding cross-sections

Holmium-166 (^166^Ho) is a β^−^ emitter that decays to ^166^Er with a half-life time of 26.8 h and emits β^−^ particles with maximum energy of 1.85 MeV (Fig. 1a). The high energy of the β^−^ particles results in a maximum tissue penetration depth of 8.7 mm which makes ^166^Ho a promising radionuclide for treating larger malignancies (Klaassen et al. 2019). In addition, ^166^Ho can also be imaged by single-photon emission computed tomography (SPECT) due to its gamma emission at 80.57 keV (Elschot et al. 2011).^166^Ho is generally produced by the neutron activation of ^165^Ho following the (n, γ) reaction. An alternative route for ^166^Ho production is the ^166^Dy/^166^Ho generator (Klaassen et al. 2019). Dysprosium-166 (^166^Dy) has a half-life time of 81.6 h, decays to ^166^Ho via β^−^ decay and can be produced by a double neutron capture reaction from ^164^Dy (Fig. 1b). ^166^Dy/^166^Ho can also serve as in vivo generator which is capable of delivering higher radiation dose per administrated activity due to the three times longer half-life time of ^166^Dy than ^166^Ho (Poty et al. 2018; Baidoo et al. 2013; Edem et al. 2016). Therefore, better treatment outcome could be expected by using ^166^Dy/^166^Ho in vivo generator instead of the direct administration of ^166^Ho.

However, Zeevaart et al. reported the radiolabelling of ^166^Dy on dodecane tetraacetic acid (DOTA) and surprisingly found that about 72% of the daughter ^166^Ho was released from the ^166^Ho-DOTA complex (Zeevaart et al. 2012). The ^166^Ho loss was attributed to the de-excitation of ^166^Ho^*^ via internal conversion instead of γ emission. Internal conversion is a process where the excited daughter nucleus electromagnetically interacts with inner orbital electrons and results in the emission of an inner electron from K shell or L shell along with the creation of electron vacancies. The electrons from the outer shells will be reorganized to fill in the vacancies while emitting auger electrons as well as characteristic X-ray. As the result of the emission of auger electrons, the de-excited ^166^Ho ions become highly charged and will extract electrons from the surrounding environment (i.e. DOTA). Due to the electron transfer to ^166^Ho, the DOTA component also becomes positively charged while the ^166^Ho ion acquires its original oxidation state (+ 3). The repulsion force between the two components having the same charge results in the rupture of the bonds between ^166^Ho and DOTA. Thus, ^166^Ho is released as free ion. The theoretical calculation predicts 73.6% ^166^Ho release which matches well with the published experimental results (Zeevaart et al. 2012). Being an isotope of a lanthanide element, free ^166^Ho tends to accumulate in liver, kidney, spleen and bone and may cause severe side effect to the patient (Suzuki et al. 1998). Therefore, to implement the ^166^Dy/^166^Ho in vivo generator in the clinic, a carrier that can prevent the loss of the internally converted ^166^Ho has to be developed.

Nowadays, the medical application of different types of nanoparticles has been extensively reported for diagnostics and the treatment of cancer and other diseases (Mitchell et al. 2021; Pelaz et al. 2017; Thomas and Weber 2019; Shi et al. 2017; Wong et al. 2020). Gold nanoparticles (AuNP) have shown great potential as carriers for anti-cancer agents due to their unique properties such as biocompatibility, precisely controlled size and the possibility of easy surface modification (Singh et al. 2018; Boisselier and Astruc 2009). Besides using AuNP as carriers for conventional payloads, multiple reports on the chelator-free labelling of medical radionuclides on AuNP have been published (Ge et al. 2020; Silva et al. 2021). In these studies, radionuclides in the form of metallic ions or halogen ions are either co-reduced into the lattice of AuNP (e.g. ^64^Cu (Frellsen et al. 2016; Sun et al. 2014; Pretze et al. 2019; Zhao et al. 2014), ^111^In (Zheng et al. 2021) and ^68^ Ga (Zheng et al. 2021)) or chemically absorbed on the surface of AuNP (^125^I, ^124^I (Lee et al. 2016; Lee et al. 2017) and ^211^At (Dziawer et al. 2017)). In most cases, the radiolabelling stability and the tumor uptake of the radionuclides appear to be improved after being loaded on AuNPs when compared to the common chelator approaches (Pretze et al. 2019). The improved tumor uptake is likely from the prolonged circulation time of AuNPs comparing with small molecules. However, the toxicity of AuNP itself have to be considered even gold is considered to be biocompatible (Ranjbar Bahadori et al. 2021).

In this study, we developed a chelator-free radiolabelling method to incorporate ^166^Dy in AuNP. In this radiolabelling method, we co-reduced ^166^Dy^3+^ ions with gold and platinum precursors to form either a bimetallic (^166^DyAuNP) or trimetallic (^166^DyPtAuNP) nanoparticle. In addition, an extra gold layer was added to the ^166^DyAuNP to form a core–shell structured ^166^DyAu@AuNP. We first characterized the physical properties of the DyAu@AuNP and DyPtAuNP with non-radioactive Dy. Then the radiolabelling of ^166^Dy was performed and the retention of ^166^Ho on ^166^DyAu@AuNP and ^166^DyPtAuNP was evaluated.

## Methods and materials

### Materials

Gold(III) chloride trihydrate (≥ 99.9%, HAuCl_4_ · 3H_2_O), chloroplatinic acid hexahydrate (≥ 37.50% Pt, H_2_PtCl_6_ · 6H_2_O), sodium borohydride (≥ 98.0%, NaBH_4_), cetyltrimethylammonium bromide (≥ 98%, CTAB), cetyltrimethylammonium chloride solution (25 wt.% in water, CTAC), L-Ascorbic acid (≥ 99%, AA), sodium hydroxide (NaOH) and dysprosium(III) chloride hexahydrate (≥ 99.9%) were purchased from Sigma-Aldrich (Zwijndrecht, the Netherlands). 90% enriched dysprosium-164 oxide powder (^164^Dy_2_O_3_) was obtained from Oak Ridge National Laboratory (sample number 122502, ORNL, Tennessee, USA). Ethylenediaminetetraacetic acid disodium salt dihydrate (Na_2_EDTA · 2H_2_O), Diethylenetriamine pentaacetate (DTPA), hydrochloric acid (HCl, 30%, Suprapur®) and nitric acid (HNO_3_, 69%, Supelco®) was supplied by Merck. All chemicals were used as received without further purification. MiliQ water was obtained from an in-house MiliQ system (Millipore) and used throughout this study.

### ***Production of ***^***166***^***Dy***

^166^Dy was produced by the double neutron capture reaction of ^164^Dy. 3 mg 90% enriched ^164^Dy_2_O_3_ powder was irradiated in the reactor facilities of the SCK•CEN—BR2 Reactor (Mol, Belgium), the Institute of Energy Security and Environmental Safety Centre for Energy Research (Budapest, Hungary) or the nuclear reactor research facility (HOR, Hoger Onderwijs Reactor) at the Department of Radiation Science and Technology of the Delft University of Technology (Delft, the Netherlands). The obtained ^166^Dy_2_O_3_ powder was dissolved in 5 ml 1 M HCl under mild heating to prepare a stock solution of ^166^DyCl_3_. 2.5 ml of the stock solution was transferred to a 20-ml glass vial and the pH of the stock solution was adjusted to ~ 5.5 by adding 2.35 ml of 1 M NaOH solution (checked by pH test paper). The activity of ^166^Dy and ^166^Ho in the stock solution was measured on a calibrated well-type HPGe detector (Canberra).

### Synthesis of AuNP seed

**Fig. 2:**
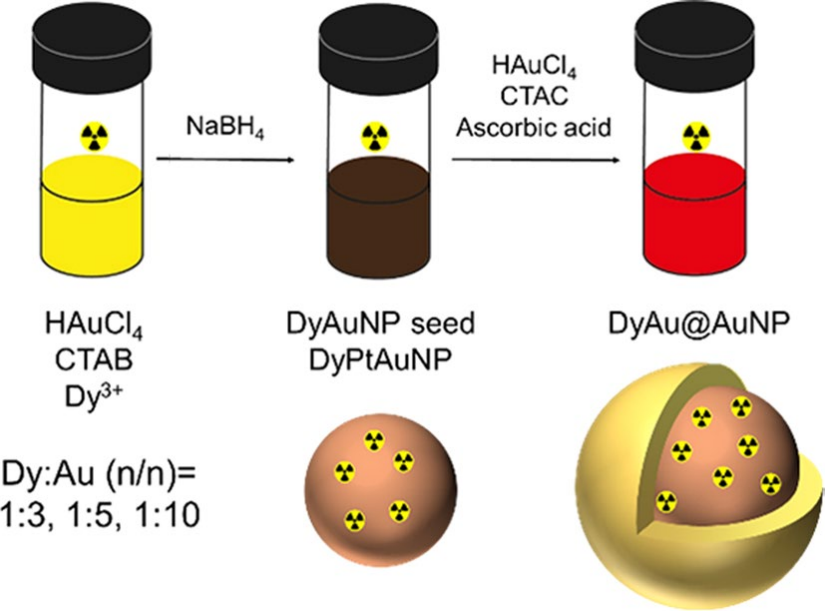
Schematic illustration of the synthesis of ^166^DyAu@AuNP and ^166^DyPtAuNP

The synthesis was adapted from a published protocol with some changes (Zheng et al. 2014) and is schematically illustrated in Fig. 2. The AuNP seeds were synthesized by the reduction of HAuCl_4_ by NaBH_4_ using CTAB as capping agent. 0.1 ml 25 mM HAuCl_4_, 4 ml 250 mM CTAB and 5.9 ml MiliQ water was added to a glass vial and mixed for 10 min. 0.6 ml freshly prepared, ice-cold 10 mM NaBH_4_ solution was added to the mixture dropwise under vigorous stirring. The color of the solution changed from yellow to dark brown rapidly. The obtained AuNP seeds were left undisturbed at 27 °C for 1.5 h before further usage.

### Growth of AuNP seed to 5 nm AuNP

2 ml 200 mM CTAC, 1.5 ml 100 mM AA and 1 ml AuNP seed dispersion were added to a glass vial and mixed for 5 min at 27 °C. 2 ml 0.5 mM HAuCl_4_ was then added in one-shot by a pipet. The reaction was continued at 27 °C for another 15 min.

### Synthesis of 5 nm non-radioactive DyAu@AuNP

33 μl, 20 μl or 10 μl 25 mM DyCl_3_ solution (pH 5.5) was mixed with 0.1 ml 25 mM HAuCl_4_ and 4 ml 250 mM CTAB in a glass vial to achieve the Dy:Au (n:n) feeding ratio of 1:3, 1:5 or 1:10. The total volume was adjusted to 10 ml by MiliQ water. 0.6 ml ice-cold 10 mM NaBH_4_ solution was then added to the mixture dropwi under vigorous stirring. The growth of the DyAuNP seed to 5 nm core–shell structured DyAu@AuNP was performed in the same way as the growth of AuNP seed to 5 nm AuNP after aging the DyAuNP seed at 27 °C for 1.5 h.

### Synthesis of non-radioactive DyPtAuNP

10 μl 25 mM H_2_PtCl_6_, 90 μl 25 mM HAuCl_4_ and 4 ml 250 mM CTAB were mixed in a glass vial. 33 μl or 10 μl 25 mM DyCl_3_ solution (pH 5.5) was then added to achieve the Dy:(Pt + Au) (n/n) feeding ration of 1:3 or 1:10. MiliQ water was added to adjust the total volume to be 10 ml and stirred for 10 min. 0.6 ml freshly prepared, ice-cold 10 mM NaBH_4_ solution was added to the mixture dropwise under vigorous stirring. The colour of the solution changed from yellow to dark brown rapidly. The obtained DyPtAuNP was left undisturbed at 27 °C for 1.5 h before purification.

### ***Synthesis of 5 nm ***^***166***^***DyAu@AuNP***

88.2 μl stock solution of ^166^DyCl_3_ containing approximately 0.134 MBq ^166^Dy and 0.2 MBq ^166^Ho was mixed with 0.1 ml 25 mM HAuCl_4_ and 4 ml 250 mM CTAB in a glass vial. 30.1 μl, 17.1 μl or 7.1 μl of 25 mM DyCl_3_ solution (pH 5.5) was then added to ensure the Dy:Au (n:n) feeding ratio to be 1:3, 1:5 or 1:10. The synthesis of ^166^DyAuNP seed and growth to ^166^DyAu@AuNP was performed in the same way as non-radioactive DyAu@AuNP which was described above.

### ***Synthesis of ***^***166***^***DyPtAuNP***

88.2 μl stock solution of ^166^DyCl_3_ containing approximately 0.134 MBq ^166^Dy and 0.2 MBq ^166^Ho was mixed with 10 μl 25 mM H_2_PtCl_6_, 90 μl 25 mM HAuCl_4_ and 4 ml 250 mM CTAB in a glass vial. 30.1 μl or 7.1 μl of 25 mM DyCl_3_ solution (pH 5.5) was then added to ensure Dy:Au (n:n) feeding ratio of 1:3 or 1:10. The final volume was then adjusted to 10 ml by MiliQ water and stirred for 10 min. 0.6 ml freshly prepared, ice-cold 10 mM NaBH4 solution was added to the mixture dropwise under vigorous stirring. The obtained ^166^DyPtAuNP was left undisturbed at 27 °C for 1.5 h before purification.

### Characterization of non-radioactive nanoparticles

The morphology and size of the AuNP, DyAu@AuNP and DyAuNP was determined with a JEM-1400 Plus transmission electron microscope (TEM, JEOL) at the acceleration voltage of 120 kV. The UV–vis absorption spectra of AuNPs were measured by a UV–VIS-NIR spectrophotometer (UV-6300PC, VWR). The hydrodynamic radius of the samples was determined by dynamic light scattering (DLS) which consisted of a JDS uniphase 633 nm 35 mW laser source, an ALV sp 125 s/w 93 goniometer, a fibre detector and a Perkin Elmer photo counter. The data was fitted using the CONTIN method and the Stokes–Einstein equation (Eq. 1) was used to determine the hydrodynamic radius of the nanoparticles.

1 $$R_{H} = \frac{kT}{{6\pi \eta D}}$$

### ***Determination of ***^***166***^***Dy radiolabelling efficiency***

100 μl 100 mM EDTA or 100 mM DTPA was added to the ^166^DyAu@AuNP and ^166^DyPtAuNP samples and incubated at 27 °C for 30 min to bind with free ^166^Dy^3+^ ions. Then the samples were centrifuged (4000 rpm, 10 min) and washed three times using spin filters (MWCO 10 KDa, Amicon). The final volume of the washed samples was adjusted to 4 ml by MiliQ water and stored at 37 °C. The counts of the nanoparticles and filtrates (^166^Dy-EDTA) of all samples were measured by an automatic gamma counter (Wallac Wizard^2^ 2480, Perkin Elmer) or a low energy Ge-detector (GL2020R, Canberra). ^166^Dy was measured using its gamma emission at 425.99 keV. The radiolabelling efficiency of ^166^Dy was calculated by the following formula: Counts(NPs)/[Counts(NPs) + ∑Counts(filtrate)] × 100%.

### ***Determination of ***^***166***^***Ho and ***^***166***^***Dy retention***

To assess the stability of ^166^Ho and ^166^Dy on nanoparticles, the samples were dispersed in 4 ml MiliQ water or 2.5 mM DTPA (pH 7.5) and incubated at 37 °C for 24, 48 and 72 h. At each time point, the samples were collected and washed by MiliQ water using spin filters under centrifugation (4000 rpm, 10 min). The counts of the nanoparticles and the filtrate was measured to calculate the retention of both ^166^Ho and ^166^Dy.

### ***Determination of gold and dysprosium content in ***^***166***^***DyAu@AuNP and ***^***166***^***DyPtAuNP***

1 ml of each completely decayed sample was completely destructed in 1 ml aqua regia (HCl/HNO_3_ = 3:1) and diluted by MiliQ water to a final volume of 10 ml. The concentration of Au and Dy were then measured by ICP-OES (Optima 8000, Perkin Elmer).

## Results

### Synthesis and characterization of non-radioactive DyAu@AuNP

In this study, we designed a core–shell structured AuNP to function as the carrier for ^166^Dy /^166^Ho in vivo generator. The gold precursor was first co-reduced with ^166^Dy^3+^ ions to form the ^166^DyAuNPs. Subsequently, an extra gold shell was grown by reducing gold precursor with ascorbic acid to prevent the possible escape of free ^166^Ho^3+^ ions. Besides assisting to retain free ^166^Ho^3+^ ions, the growth of an extra gold layer can also improve the colloidal stability of the DyAuNPs (Zheng et al. 2014). It is important that the original physiochemical properties of the AuNPs are not altered upon ^166^Dy encapsulation. Thus, we first performed a pilot study with non-radioactive DyCl_3_ to explore the influence of Dy content on the physical properties of the DyAu@AuNPs. The non-radioactive DyCl_3_ was co-reduced with HAuCl_4_ by a strong reducing agent NaBH_4_ to form the bimetallic DyAuNPs. Three samples with different Dy:Au feeding ratios of 1:3, 1:5 and 1:10 were prepared. An extra gold shell was then grown on the seed particles via the reduction of HAuCl_4_ at lower concentration using ascorbic acid and resulting in the core–shell structured DyAu@AuNPs. The non-incorporated Dy^3+^ ions were removed by incubating DyAu@AuNPs with EDTA or DTPA, followed by multiple cycles of washing with MiliQ water. Au@AuNP without Dy content was also prepared with the same method and used as the control group.

**Fig. 3:**
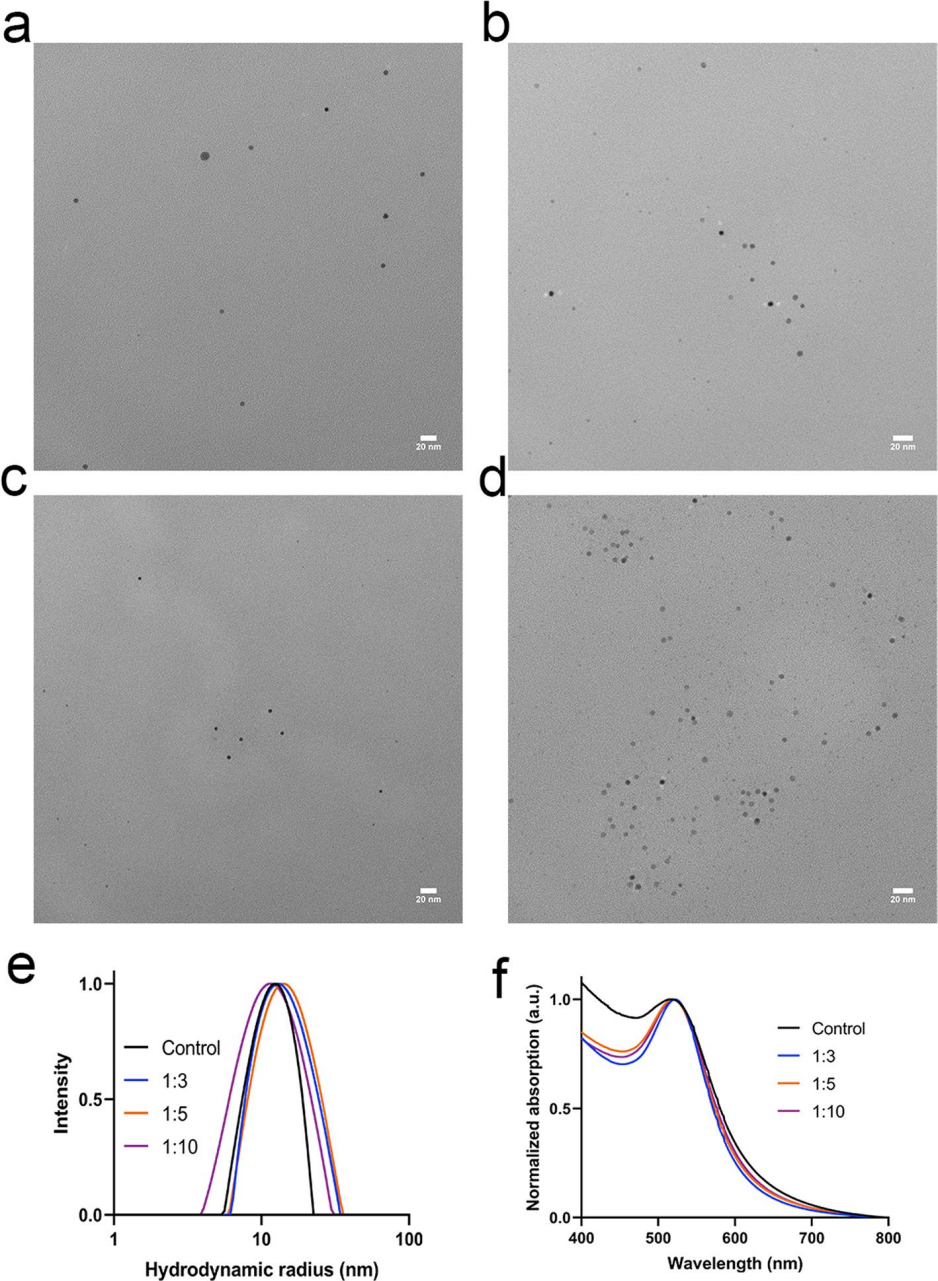
Characterization of DyAu@AuNPs. **a**–**d** Representative TEM image of samples with different Dy:Au feeding ratios: Dy:Au = 1:3 (**a**), 1:5 (**b**), 1:10 (**c**), no Dy addition (**d**). Scale bar is 20 nm. See supporting information for size distribution histograms (Additional file 1: Fig. S1). **e** Hydrodynamic radius (R_H_) of the samples measured by DLS. f) UV–vis spectrum of the DyAu@AuNPs

The size and shape of the DyAu@AuNPs were characterized by transmission electron microscope (TEM). As shown in Fig. 3a–d, DyAu@AuNPs with varying Dy:Au feeding ratios as well as the Au@AuNP all showed a diameter of 4.9 nm. The hydrodynamic radius (R_H_) of the DyAu@AuNPs and Au@AuNP was measured by dynamic light scattering (DLS). As shown in Fig. 3e, the intensity weighted R_H_ was determined to be within the range of 12 ~ 14 nm for both the DyAu@AuNPs and the Au@AuNP. The hydrodynamic radius of the DyAu@AuNPs was found to be larger than the radius measured by TEM, since DLS measures the hydration layer formed around CTAB/CTAC on the surface of the nanoparticles. Due to the surface plasmon resonance (SPR) effect of AuNPs, the characteristic UV–vis spectrum can be used as an indication of the size of AuNPs (Barbosa et al. 2010). The UV–vis spectrum of the DyAu@AuNPs and Au@AuNP is shown in Fig. 3f. The wavelength of the SPR peak (λ_SPR_) of all samples were detected near 520 nm, indicating that the DyAu@AuNPs all had comparable size to the Au@AuNP. All results of the characterization of the DyAu@AuNPs and Au@AuNP are summarized in Table 1.

**Table 1:** Summary of the physical properties of DyAu@AuNPs with different Dy:Au feeding ratios

	No Dy	1:3	1:5	1:10
d (nm)	4.9 ± 0.8	4.9 ± 0.6	4.9 ± 0.7	4.9 ± 0.7
R_H_ (nm)	12.3 ± 0.3	12.9 ± 0.3	14.1 ± 0.5	11.8 ± 0.3
λ_SPR_ (nm)	518	522	519	520

Based on the TEM, UV–vis and DLS measurements, we conclude that incorporating different amounts of Dy into the gold nanoparticle had no influence on the final size and shape of the core–shell structured DyAu@AuNPs.

### Synthesis and characterization of non-radioactive DyPtAuNP

**Fig. 4:**
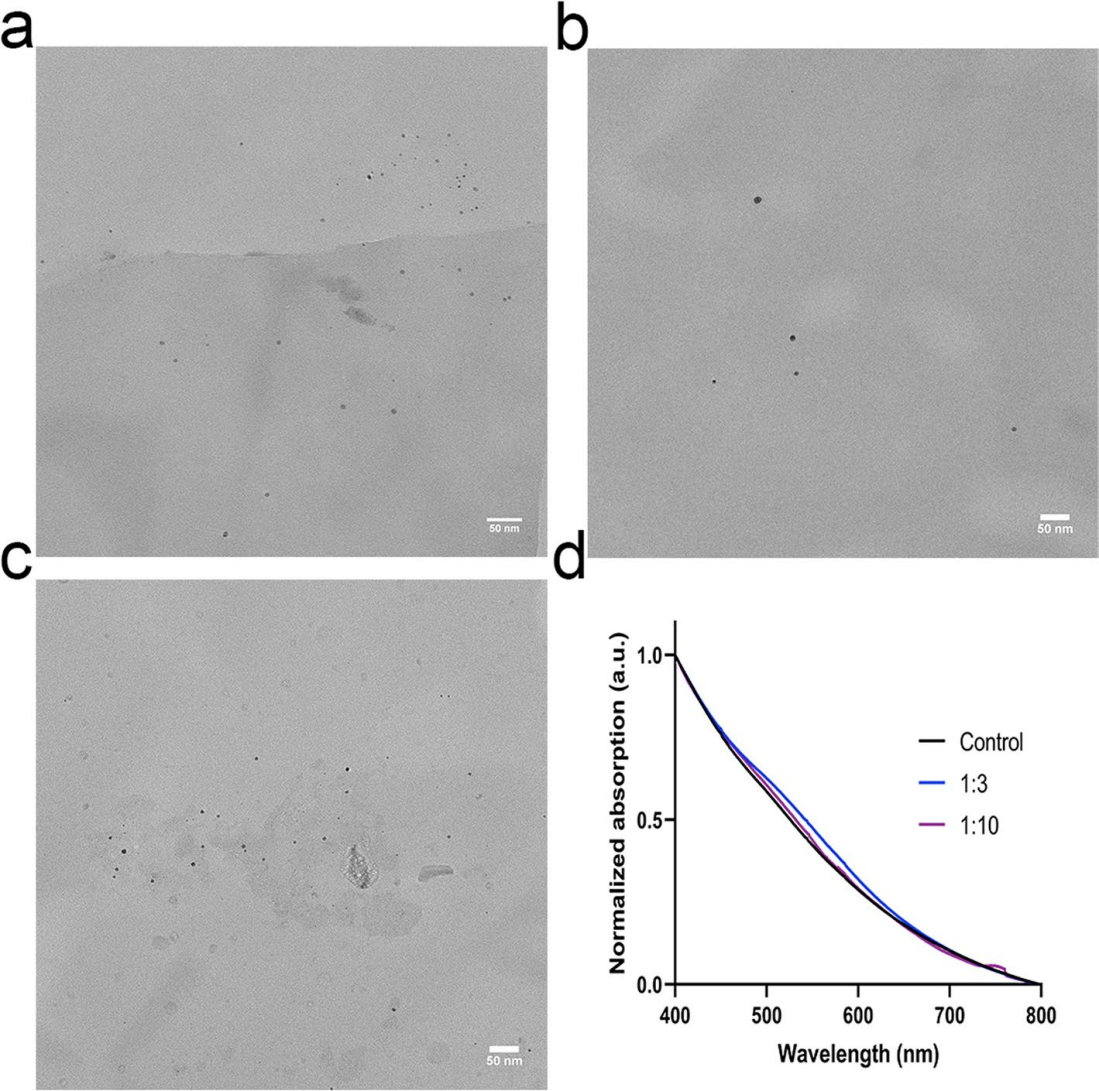
Characterization of DyPtAuNPs. **a**–**c** Representative TEM image of samples with different Dy:Au feeding ratios: Dy:Au = 1:3 (**a**), 1:10 (**b**) and no Dy addition (**c**). Scale bar is 50 nm. See supporting information for size distribution histograms (Additional file 1: Fig. S2). **d** UV–vis spectrum of the DyPtAuNPs

To better understand the behavior of ^166^Ho and ^166^Dy on nanoparticles and check if internally converted ^166^Ho can be retained even without the extra gold layer, we attempted to directly use the DyAuNPs as the carrier for ^166^Dy. However, the DyAuNPs were not stable and aggregated to larger AuNPs within 24 h (Additional file 1: Fig. S3). To improve the colloidal stability of DyAuNPs, we hereby prepared trimetallic DyPtAuNPs by replacing 10% of Au with Pt while the Dy:(Au + Pt) feeding ratios was still set to be 1:3 and 1:10. PtAuNP with no Dy content was also prepared and used as control. The size of the DyPtAuNPs as determined by TEM are shown in Fig. 4a–c. The diameter of the DyPtAuNP with Dy feeding ratio of 1:3 was measured to be 4.4 ± 1.1 nm which is comparable to the PtAuNP (4.0 ± 1.3 nm). However, larger particles (d = 6.6 ± 1.8 nm) were measured for the DyPtAuNP with Dy feeding ratio of 1:10. The UV–vis spectrum of the DyPtAuNPs and PtAuNP is given in Fig. 4d. The absence of SPR peak near 500 nm further confirmed the small size of the nanoparticles (Alric et al. 2013).

## Radiolabelling of ^166^Dy on DyAu@AuNP and DyPtAuNP

**Fig. 5:**
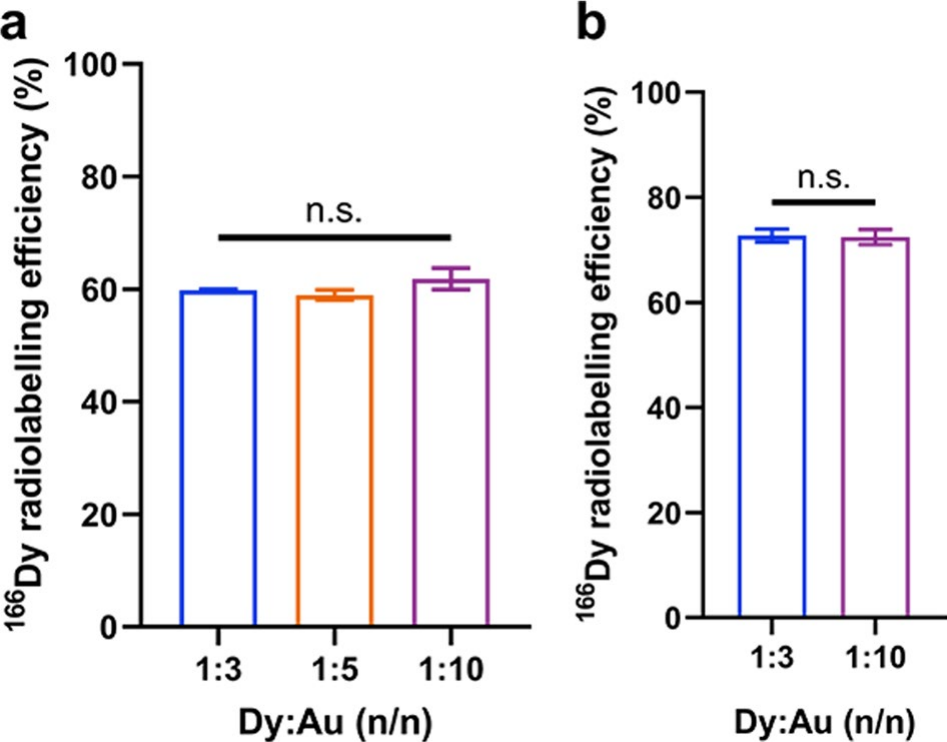
^166^Dy radiolabelling efficiency of ^166^DyAu@AuNP (**a**) and ^166^DyPtAuNP (**b**) with different Dy:Au feeding ratios. The error bars represent the standard deviations of three independent experiments ( n.s. indicates non-significant difference, 2way ANOVA test)

The radiolabelling of ^166^Dy was carried out by a similar method as used for the preparation of non-radioactive DyAu@AuNPs and DyPtAuNPs. The Dy source was changed to a mixture of non-radioactive DyCl_3_ and ^166^DyCl_3_ stock solution containing 0.134 MBq ^166^Dy. Due to the decay of ^166^Dy, ^166^Ho was also present in the stock solution of ^166^DyCl_3_. Considering the trace amount of ^166^Ho^3+^ ions, we expect that this to have negligible influence on the formation of the NPs. Three independent samples of ^166^DyAu@AuNP and ^166^DyPtAuNP with different Dy:Au feeding ratios were prepared and washed thoroughly by EDTA/DTPA and MiliQ water to remove all unbounded ^166^Dy. The ^166^Dy radiolabelling efficiency was calculated by comparing the counts of nanoparticles and the washing solution at 425.99 keV. The calculated results are shown in Fig. 5. Radiolabelling efficiency of 60% and 70% was achieved for ^166^DyAu@AuNPs and ^166^DyPtAuNPs respectively. No significant difference of the radiolabelling efficiency was found among the groups with different Dy:Au feeding ratios.

Due to the big lattice mismatch (11.9%) and the large difference of reduction potential between Dy (III, − 2.29 V) and Au (III, [AuCl_4_]^−^, + 0.93 V), not all initially added Dy was reduced in the AuNP core which resulted in ^166^Dy radiolabelling efficiency of around 60%. As the same activity of ^166^DyCl_3_ was used during the synthesis of ^166^DyAu@AuNPs and ^166^DyPtAuNPs, the activity of radiolabelled ^166^Dy was the same for all samples with different Dy:Au feeding ratios. No improvement of the ^166^Dy radiolabelling efficiency was achieved by lowering the initial amount of Dy^3+^.

The completely decayed ^166^DyAu@AuNP and ^166^DyPtAuNP samples were also destructed and further analysed by ICP-OES to measure the concentration of Au and Dy. Comparing with the Au concentration of the Au@AuNP and PtAuNP samples, little difference of the Au concentration was found from the ^166^DyAu@AuNP and ^166^DyPtAuNP samples (Table S1). Taking ICP-OES measurements together with other characterizations, we further confirmed that the reduction of gold precursor by NaBH_4_ as well as the formation of nanoparticles was not affected by the addition of Dy^3+^. The ^166^Dy radiolabelling efficiency was also calculated using the total concentration of Dy (including both radioactive and non-radioactive Dy) measured by ICP-OES (Additional file 1: Fig. S4). Similar to the results from Ge-detector measurement, the radiolabelling efficiency was not influenced by the Dy:Au feeding ratios. However, we found that the ^166^Dy radiolabelling efficiency calculated from ICP-OES data was approximately 10% lower than that from the Ge-detector data. Further studies will be carried out to explain this phenomenon.

### ***Retention of ***^***166***^***Ho and ***^***166***^***Dy***

In vivo generator of therapeutic radionuclides can generally increase the delivered dose per administrated activity because of the longer half-life time of the mother nuclides (Edem et al. 2016). To make sure the radiation dose is mainly delivered to the tumor while sparing the normal tissues, both the mother and the daughter nuclides should be kept within the carrier. Therefore, we radiolabelled core–shell structured gold nanoparticles, i.e. the ^166^DyAu@AuNPs with ^166^Dy. An outer layer of gold was added to prevent the diffusion of free ^166^Ho if it escapes from the core nanoparticle. On the other hand, nanoparticles without the shell structure, i.e. the ^166^DyPtAuNPs were also radiolabelled with ^166^Dy for comparison. ^166^DyAuNP seeds were not studied because of the low colloidal stability (Additional file 1: Fig. S3).

**Fig. 6:**
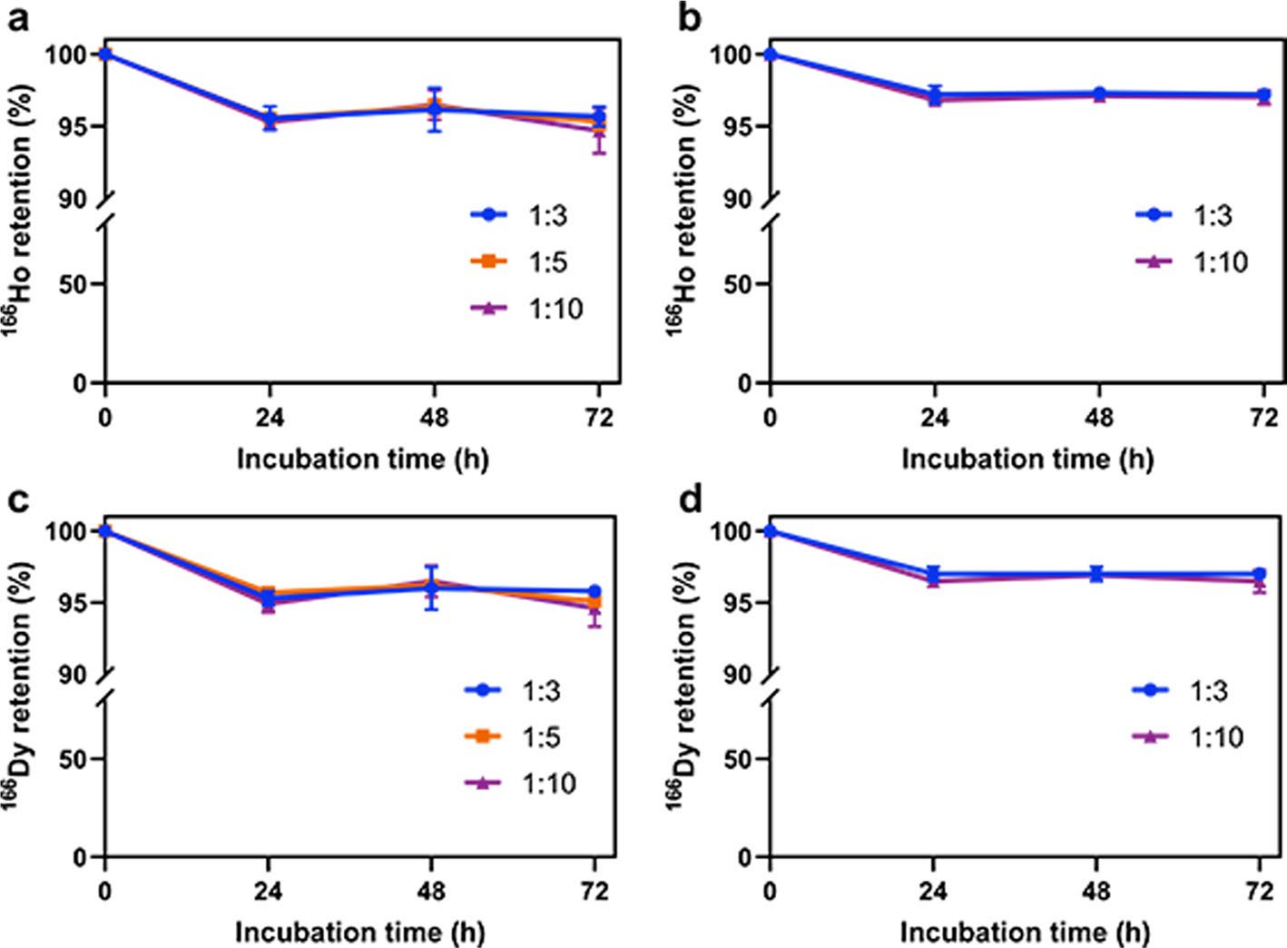
^166^Ho and ^166^Dy retention of ^166^DyAu@AuNPs (**a**, **c**) and ^166^DyPtAuNPs (**b**, **d**) with different Dy:Au feeding ratios in MiliQ water at 37 °C as function of time. The error bars represent the standard deviation of three independent experiments

**Fig. 7:**
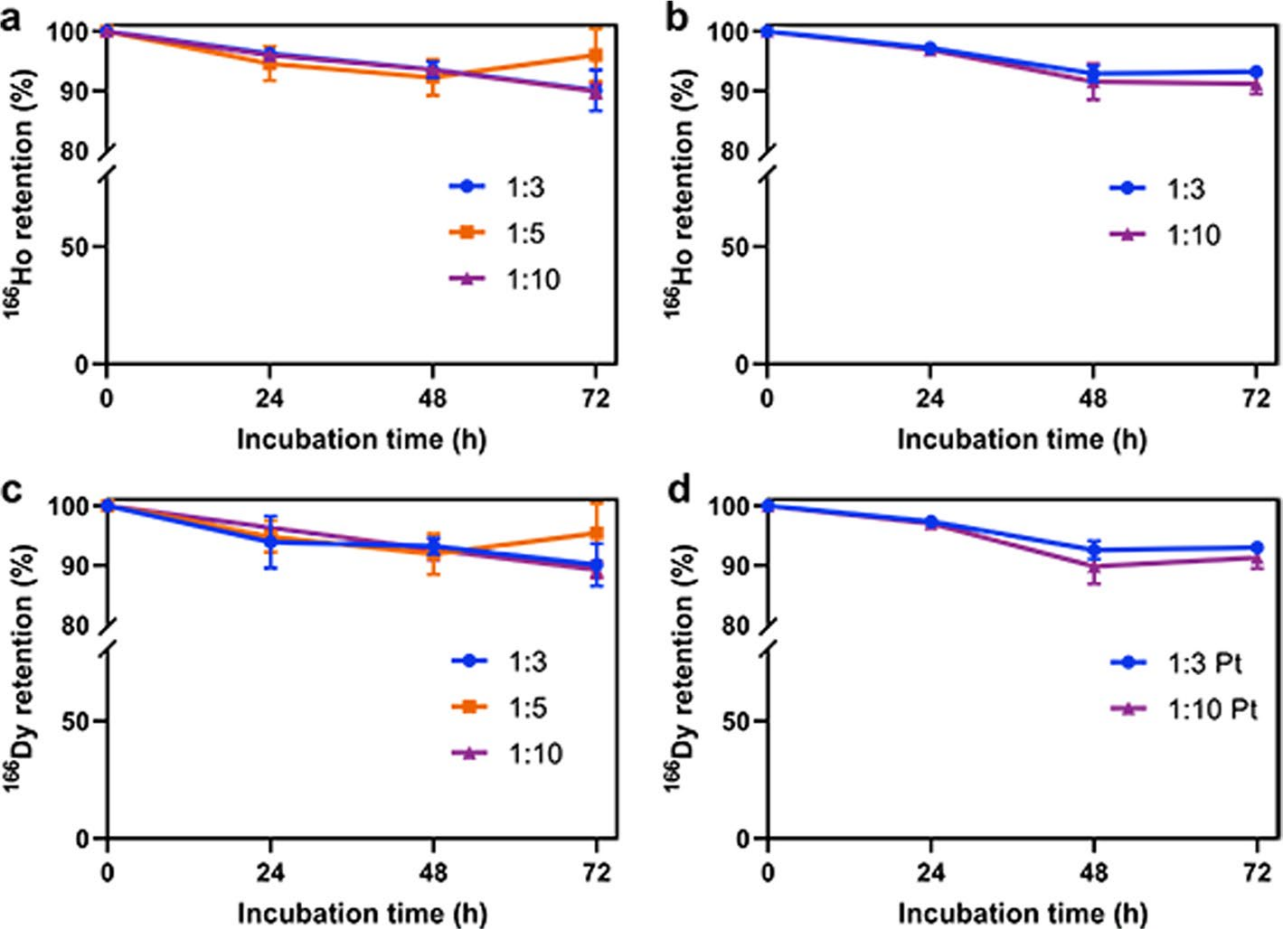
^166^Ho and ^166^Dy retention of ^166^DyAu@AuNPs (**a**, **c**) and ^166^DyPtAuNPs (**b**, **d**) with different Dy:Au feeding ratios in 2.5 mM DTPA at 37 °C as function of time. The error bars represent the standard deviation of three independent experiments

To measure the retention of the internally converted ^166^Ho as well as the retention of ^166^Dy, ^166^DyAu@AuNPs and ^166^DyPtAuNPs were incubated in MiliQ water or 2.5 mM DTPA (pH 7.5) at 37 °C for 72 h. Every 24 h, the samples were centrifuged to separate NPs from free ^166^Dy^3+^ and ^166^Ho^3+^. The counts of the nanoparticles and the washing solution was measured at 65 ~ 90 keV and 340 ~ 460 keV for ^166^Ho and ^166^Dy respectively. As the NPs were still capped by CTAB/CTAC, the nanoparticles would form aggregation upon interaction with high concentration salt solution or protein (Zhang and Lin 2014). Thus, the in vitro stability tests were not performed in PBS or serum to avoid the interference of nanoparticle aggregation. As shown in Fig. 6, more than 95% of ^166^Ho was found to be retained in both ^166^DyAu@AuNPs and ^166^DyPtAuNPs for at least 72 h in MiliQ water (Fig. 6a, b). The retention of ^166^Dy was also found to be more than 95% for both ^166^DyAu@AuNPs and ^166^DyPtAuNPs during the 72 h incubation in MiliQ water (Fig. 6c, d). For all the samples challenged by DTPA, about 90% of both ^166^Ho and ^166^Dy was still bounded to the nanoparticles even after 72 h incubation (Fig. 7). These results indicate that very high ^166^Ho and ^166^Dy retention was achieved independent from the Dy:Au feeding ratio and the extra shell of coating.

## Discussion

Surprisingly, the ^166^DyPtAuNPs were found to be able to retain the same percentage of ^166^Ho as the ^166^DyAu@AuNPs. This result suggests that high ^166^Ho retention could still be achieved even without the addition of an extra gold layer. This finding made us think about a the possible mechanism responsible for the high ^166^Ho retention on AuNPs. The internal conversion of ^166^Dy results in highly charged ^166^Ho ions which tend to seek electrons from the surrounding environment, i.e. the carrier. In the case of ^166^Dy coupled to a simple chelator composed of low Z elements such as dodecane tetraacetic acid (DOTA), the number of free electrons in the system is low. Therefore, DOTA molecule could be easily altered to be positively charged after the electron migration to the ^166^Ho ions. Due to the repulsion between the entities having the same charge, i.e. ^166^Ho^3+^ and [DOTA]^n+^, the ^166^Ho-DOTA complex is ruptured. When a high Z material is used as the carrier for ^166^Dy, such as AuNP, many more free electrons are available. When the highly positive ^166^Ho ion extracts electrons from its neighbouring Au atoms, electrons can be quickly redistributed to fill in the new vacancies. The redistribution of electrons might cause a transient change of the surface charge of AuNP, but then electrons from the solvent (i.e. water) will be attracted to the AuNP due to the ultra-high affinity of Au to solvated electrons (Ghandi et al. 2015). Therefore, the colloidal stability of AuNP is preserved while the release of ^166^Ho is avoided. A similar method was reported to improve the retention of ^80^Br which was internally converted from ^80m^Br (49 and 37 keV, α = 1.6 and 300 respectively) by Adamson et al. (Adamson and Grunland 1951; Wexler and Anderson 1960). The authors found that 100% and 86% of ^80^Br was released from [Co(NH_3_)_5_Br]^2+^ (aq) and solidified [Co(NH_3_)_5_Br](NO_3_)_2_ (s) while 47% and even 0% of ^80^Br was released from [PtBr_6_]^2−^ (aq) and solidified (NH_4_)_2_PtBr_6_ (s). This result supported our hypothesis on the function of AuNP as electron source for the internally converted ^166^Ho. Besides, the results from these studies also suggest that in our case the reduction of ^166^Dy^3+^ into solid state (Dy^0^) might also contribute to the high retention of ^166^Ho.

Besides the high retention of the internally converted ^166^Ho, our radiolabelling method is also simple and quick. The whole procedure can be finished within 8 h without the need of separating ^166^Dy from ^166^Ho. The interaction between the β^−^ particle emitted by ^166^Ho and gold atoms is also favourable for a more efficient dose delivery due to the formation of secondary electrons and free radicals such as · OH radicals (Haume et al. 2016). To make the ^166^DyPtAuNPs and ^166^DyAu@AuNPs more applicable for clinical application, the current capping ligand, CTAB/CTAC, has to be exchanged with biocompatible ligands such as PEG. In previous studies it has been shown that small AuNPs not conjugated with targeting agents have tumour uptake around 4–5% ID/g depending on the morphology and surface properties of the nanoparticles (Sun et al. 2014; Zhang et al. 2022). In comparison small molecules such as PSMA can achieve much higher tumour uptake (Banerjee et al. 2014). Therefore, it will be very interesting to determine whether the addition of such targeting moieties will increase tumour accumulation.

## Conclusion

In summary, we developed a chelator-free radiolabelling method to obtain a ^166^Dy/^166^Ho in vivo generator and prevented the loss of ^166^Ho that is caused by internal conversion. The explanation for the high ^166^Ho retention was not experimentally proven but might be related to the high electron density of the gold nanoparticles. To further understand the mechanism of ^166^Ho retention on gold nanoparticles, the structure of the nanoparticles should be studied by both experiments as well as theoretical simulations. Besides the further research on ^166^Ho retention mechanism, the capping ligands of the nanoparticles should be replaced to increase the biocompatibility of the nanoparticles and make them suitable for medical applications.

## Supplementary Information

**Additional file1**. Theoretical calculation of ^166^Ho loss due to internal conversion; **Fig. S1**. Size distribution histogram of DyAu@AuNPs with different Dy:Au feeding ratios; **Fig. S2**. Size distribution histogram of DyPtAuNPs with different Dy:Au feeding ratios; **Fig. S3**. Representative picture of ^166^DyAuNP (Dy:Au=1:3) after 24 h incubation at 37  °C; **Fig. S4**. Comparison of ^166^Dy radiolabelling efficiency calculated from Ge-detector data and ICP-OES data; **Table S1**. Comparison of Au concentration of Au@AuNP, ^166^DyAu@AuNP, PtAuNP and ^166^DyPtAuNP

## Data Availability

The data associated to this research work are available in this manuscript or in the online supplementary file.

## Abbreviations

AA: Ascorbic acid
AuNP: Gold nanoparticle
CTAB: Cetyltrimethylammonium bromide
CTAC: Cetyltrimethylammonium chloride
DLS: Dynamic light scattering
DOTA: Dodecane tetraacetic acid
DTPA: Diethylenetriamine pentaacetate
Dy: Dysprosium
EBRT: External beam radiation therapy
EDTA: Ethylenediaminetetraacetic acid
Ho: Holmium
RNT: Radionuclide therapy
SPECT: Single photon emission computed tomography
SPR: Surface plasmon resonance
TEM: Transmission electron microscope
UV–vis: Ultraviolet–visible
