# Supplementary material for: Core–shell structured gold nanoparticles as carrier for 166Dy/166Ho in vivo generator
Source: EJNMMI Radiopharm Chem. 2022 Jul 19;7:16. doi: 10.1186/s41181-022-00170-3 (PMC9296738; doi:10.1186/s41181-022-00170-3)
Supplement: Supplementary file 1 — Additional file1. Theoretical calculation of 166Ho loss due to internal conversion; Fig. S1. Size distribution histogram of DyAu@AuNPs with different Dy:Au feeding ratios; Fig. S2. Size distribution histogram of DyPtAuNPs with different Dy:Au feeding ratios; Fig. S3. Representative picture of 166DyAuNP (Dy:Au=1:3) after 24 h incubation at 37 °C; Fig. S4. Comparison of 166Dy radiolabelling efficiency calculated from Ge-detector data and ICP-OES data; Table S1. Comparison of Au concentration of Au@AuNP, 166DyAu@AuNP, PtAuNP and 166DyPtAuNP [file 41181_2022_170_MOESM1_ESM.docx]

**Core-shell structured gold nanoparticles as carrier for**

**^166^Dy/^166^Ho in vivo generator**

Runze Wang^1^, Bernard Ponsard^2^, Hubert Wolterbeek^1^ & Antonia Denkova^1, *^

^1^*Applied Radiation and Isotopes, Department of Radiation Science and Technology, Faculty of Applied Sciences, Delft University of Technology, Mekelweg 15, 2629 JB, Delft, the Netherlands*

^2^*BR2 Reactor, Belgian Nuclear Research Centre, SCK CEN, Boeretang 200, B-2400, Mol, Belgium*

^*^Corresponding author: Antonia Denkova

E-mail addresses: A.G.Denkova@tudelft.nl

Study design:

In this paper, we developed an nanoparticle based carrier for the ^166^Dy/^166^Ho in vivo generator. By radiolabelling ^166^Dy on the carriers designed in this work, much higher retention of the daughter nuclide ^166^Ho (>90%) was achieved comparing with the conventional ^166^Dy-DOTA complex (28%) as reported by others. The reason for this dramatic enhancement of ^166^Ho was also discussed. Overall speaking, a general method to avoid the loss of daughter nuclides caused by the chemical effects after the decay of the mother nuclides (mainly internal conversion) was proposed in this paper.

**Table of contents**

- Theoretical calculation of ^166^Ho loss due to internal conversion
- Figure S1. Size distribution histogram of DyAu@AuNPs with different Dy:Au feeding ratios
- Figure S2. Size distribution histogram of DyPtAuNPs with different Dy:Au feeding ratios
- Figure S3. Representative picture of ^166^DyAuNP (Dy:Au=1:3) after 24 h incubation at 37 ℃
- Figure S4. Comparison of ^166^Dy radiolabelling efficiency calculated from Ge-detector data and ICP-OES data
- Table S1. Comparison of Au concentration of Au@AuNP, ^166^DyAu@AuNP, PtAuNP and ^166^DyPtAuNP

**Theoretical calculation of ^166^Ho loss due to internal conversion**

The possibility of internal conversion and emission of gamma rays can be described by a factor called internal conversion coefficient (α).

$$\alpha=\frac{number of de-excitations by the release of conversion electrons}{number of de-excitations via gamma ray emission}$$

In the case of ^166^Dy, its emission at 82.5 keV has an overall internal conversion coefficient of 4.02 which indicates that about 80% of this transition is by internal conversion^1^.

$$Probability of internal conversion=\frac{\alpha}{1+\alpha}$$

Taking the branching ratio of this transition line (92.5%) into account, the probability of internal conversion of the β^-^ decay of ^166^Dy can be calculated by 80% × 92.5%=73.6%.





Figure S1. Size distribution histogram of DyAu@AuNPs with different Dy:Au feeding ratios: No addition of Dy (a), Dy:Au=1:3 (b), 1:5 (c) and 1:10 (d).


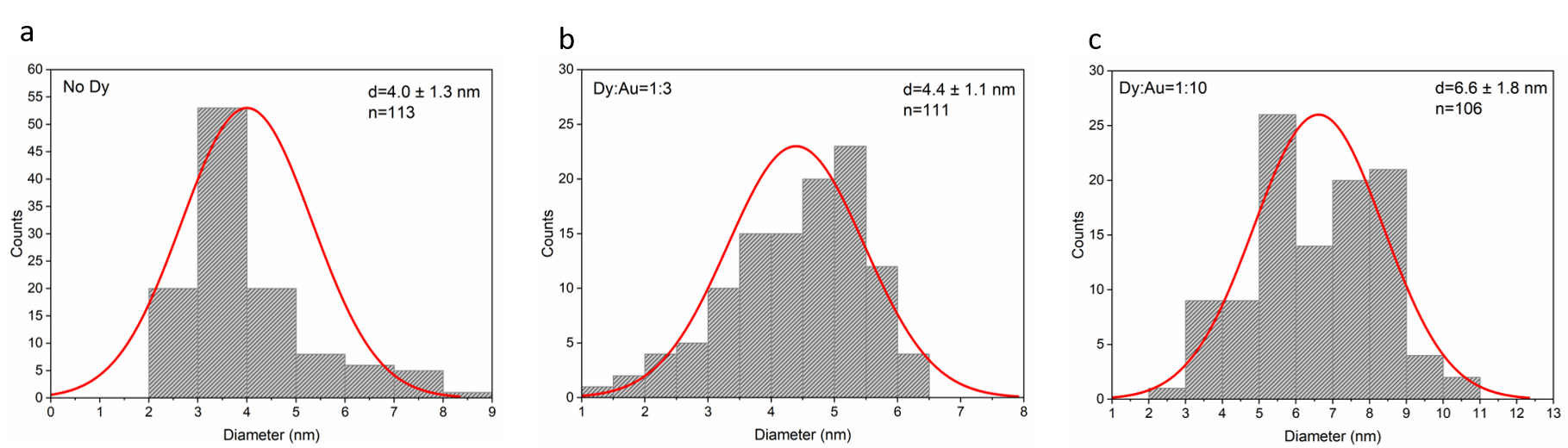


Figure S2. Size distribution histogram of DyPtAuNPs with different Dy:Au feeding ratios: No addition of Dy (a), Dy:Au=1:3 (b) and 1:10 (c).


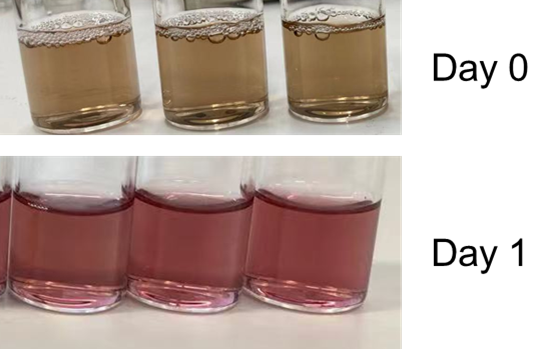


Figure S3. Representative picture of ^166^DyAuNP (Dy:Au=1:3) after 24 h incubation at 37 ℃. The color change from dark brown to purple red is an indication of change of the nanoparticle size. To avoid the interference of the nanoparticle size change on the ^166^Ho retention and ^166^Dy radiostability, the ^166^DyAuNPs were not further studied in this work.


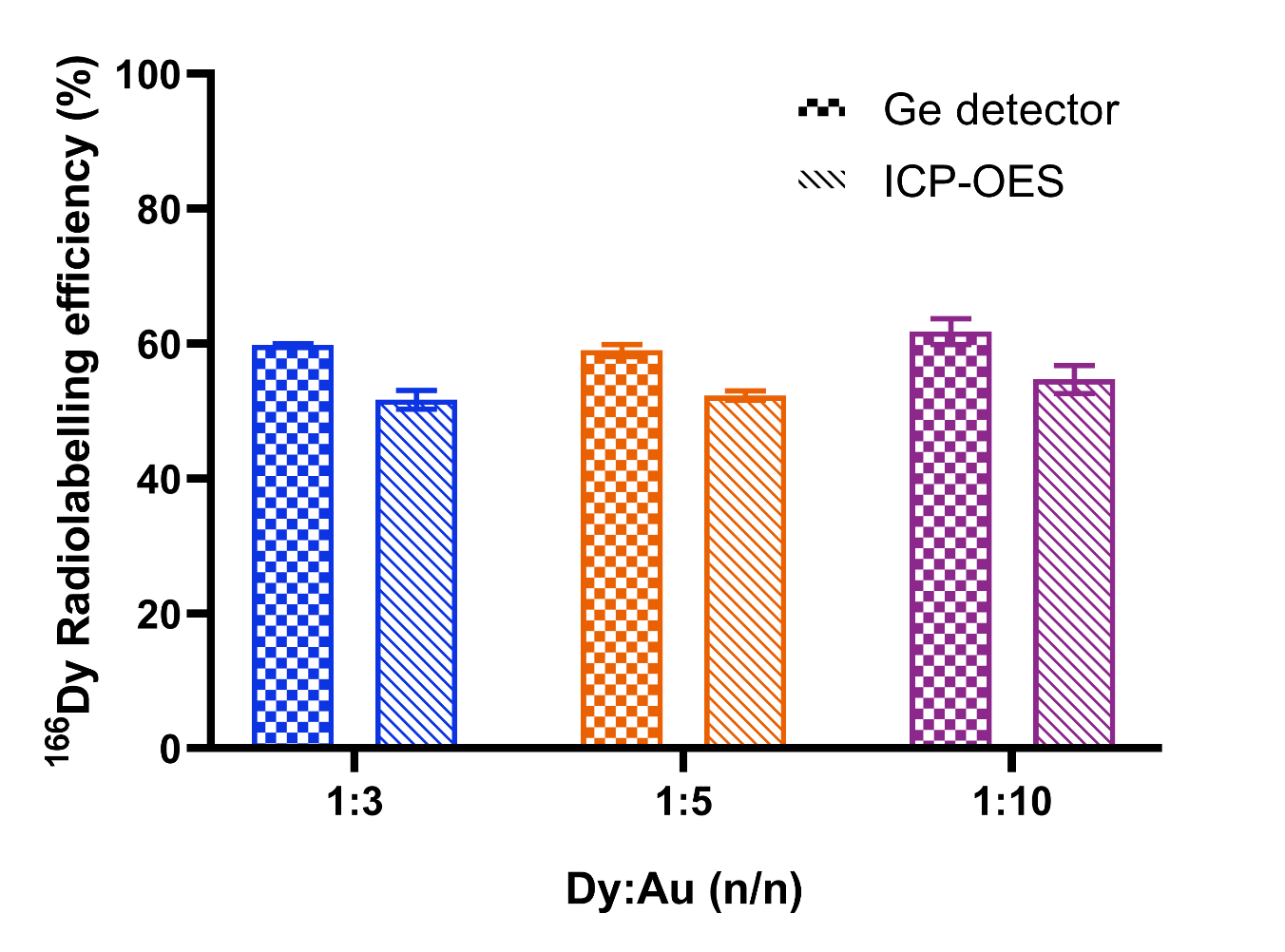


Figure S4. Comparison of ^166^Dy radiolabelling efficiency calculated from Ge-detector data (dot) or ICP-OES (line) data. The ICP-OES data based ^166^Dy radiolabelling efficiency was found to be about 10% lower than that calculated using Ge-detector data.

Table S1. Comparison of Au concentration among Au@AuNP, ^166^DyAu@AuNP, PtAuNP and ^166^DyPtAuNP. The Au concentration was measured by ICP-OES after the complete decay of ^166^Dy.

|  | Dy:Au feeding ratio | [Au] mg/L^*^ |
| --- | --- | --- |
| Au@AuNP | - | 109.7 |
| ^166^DyAu@AuNP | 1:3 | 115.1 |
| ^166^DyAu@AuNP | 1:5 | 109.7 |
| ^166^DyAu@AuNP | 1:10 | 110.1 |
|  | | |
| PtAuNP | - | 93.9 |
| ^166^DyPtAuNP | 1:3 | 89.5 |
| ^166^DyPtAuNP | 1:10 | 91.5 |

*The [Au] concentration for core-shell structured NPs and PtAuNPs are all based on a final volume of 4 ml after washing.
